# Influence of Soft and Stiff Matrices on Cytotoxicity in Gingival Fibroblasts: Implications for Soft Tissue Biocompatibility

**DOI:** 10.3390/cells13231932

**Published:** 2024-11-21

**Authors:** Ye-Jin Yang, Donghyeon Yeo, Seong-Jin Shin, Jun Hee Lee, Jung-Hwan Lee

**Affiliations:** 1 Institute of Tissue Regeneration Engineering (ITREN), Dankook University, 119 Dandae-ro, Cheonan 31116, Republic of Korea; yejin7068@naver.com (Y.-J.Y.); d72220336@dankook.ac.kr (D.Y.); ko2742@naver.com (S.-J.S.); junheelee@dankook.ac.kr (J.H.L.); 2 Department of Biomaterials Science, College of Dentistry, Dankook University, 119 Dandae-ro, Cheonan 31116, Republic of Korea; 3 Department of Nanobiomedical Science & BK21 FOUR NBM Global Research Center for Regenerative Medicine, Dankook University, 119 Dandae-ro, Cheonan 31116, Republic of Korea; 4 Mechanobiology Dental Medicine Research Center, Dankook University, 119 Dandae-ro, Cheonan 31116, Republic of Korea; 5 UCL Eastman-Korea Dental Medicine Innovation Centre, Dankook University, 119 Dandae-ro, Cheonan 31116, Republic of Korea; 6 Cell & Matter Institute, Dankook University, 119 Dandae-ro, Cheonan 31116, Republic of Korea

**Keywords:** human gingival fibroblasts, substrate stiffness, methyl methacrylate, cytotoxicity, biocompatibility assessment

## Abstract

The biocompatibility of dental materials is critical for ensuring safety in clinical applications. However, standard in vitro cytotoxicity assays often rely on stiff tissue culture plastic (TCP), which does not accurately replicate the biomechanical properties of soft oral tissues. In this study, we compared human gingival fibroblasts (HGFs) cultured on soft, gel-based substrates mimicking gingival tissue stiffness (0.2 kPa) with those cultured on conventional TCP (3 GPa) to assess the influence of substrate stiffness on the cytotoxicity of methyl methacrylate (MMA), as well as other cytotoxic agents, including DMSO and H_2_O_2_. The results demonstrated that cells cultured on softer substrates exhibited enhanced resistance to cytotoxic stress, with increased viability and decreased apoptosis and DNA damage following exposure to MMA, DMSO, and H_2_O_2_. Notably, HGFs on soft substrates showed significantly greater resilience to MMA-induced cytotoxicity compared to those cultured on TCP. These findings emphasize the critical role of substrate stiffness in modulating cellular responses to toxic agents and highlight the necessity of using physiologically relevant models for cytotoxicity testing of dental materials. This study provides valuable insights for improving biocompatibility assessment protocols in clinical settings.

## 1. Introduction

The biocompatibility of biomaterials, including dental materials, is essential for ensuring their safety and effectiveness in clinical application [1,2,3,4,5]. ISO Standard 10993 mandates comprehensive biocompatibility testing during material development [6,7,8], while ISO 7405 provides specific guidelines for evaluating dental materials [9]. A range of studies has investigated the cytotoxicity of dental materials using various models, including in vitro cell-based assays, animal studies, and clinical trials [10,11,12]. Among these, in vitro cell-based assays are most frequently used for initial screening due to their efficiency and cost-effectiveness [13,14]. These assays offer critical preliminary data on material interactions with biological tissues, particularly in the context of dental resins and adhesives that come into contact with soft tissues such as the gingiva.

Although in vitro cytotoxicity assays are widely used, concerns have been raised about using stiff tissue culture plastic (TCP), as mandated by ISO guidelines, which may not accurately replicate the physiological conditions of soft tissues [15,16]. This is particularly relevant given emerging evidence across diverse fields demonstrating how substrate mechanics profoundly impact cellular behavior. For instance, studies in mechanobiology have shown that matrix stiffness directs stem cell fate, with soft matrices promoting neurogenic differentiation while stiff substrates favor osteogenesis [17,18]. Similarly, in cancer biology, matrix rigidity has been found to modulate drug resistance and cellular survival [19,20], while in tissue regeneration, substrate mechanics influence fibroblast activation and extracellular matrix remodeling [21,22]. These insights into mechanotransduction raise critical questions about the relevance of current biocompatibility testing methods.

Indeed, TCP, with a stiffness of approximately 3 GPa, differs significantly from biological tissues like gingiva, which have a much lower elastic modulus. This mechanical disparity raises concerns about the relevance of cytotoxicity data obtained from these models. Studies have shown that the physical properties of cell culture substrates, such as stiffness and dimensionality, can significantly influence cellular responses to toxic agents [23,24,25]. For instance, cells cultured in three-dimensional (3D) environments generally show reduced sensitivity to toxins compared to those grown on two-dimensional (2D) substrates [26,27,28]. Additionally, materials like composite resins, Intermediate Restorative Material (IRM), and Zinc Oxide Eugenol (ZOE), which are widely used in clinical dentistry for their restorative properties, are generally considered biocompatible in vivo. IRM, a zinc oxide-based material reinforced with polymethyl methacrylate, and ZOE, a mixture of zinc oxide and eugenol, are particularly valued for their compatibility with soft tissues in temporary restorations. However, these materials often exhibit cytotoxic effects when tested on TCP [29,30,31]. These inconsistencies likely result from differences between the soft, compliant in vivo environment and the stiff in vitro conditions. This discrepancy highlights the need to refine in vitro testing methods to better mimic physiological environments. To improve the accuracy of our assays, we synchronized the cells to the same cell cycle phase through starvation, ensuring consistent conditions for evaluating cytotoxicity. This refinement aimed to reduce variability in cellular responses during toxic agent exposure.

**Figure 1:**
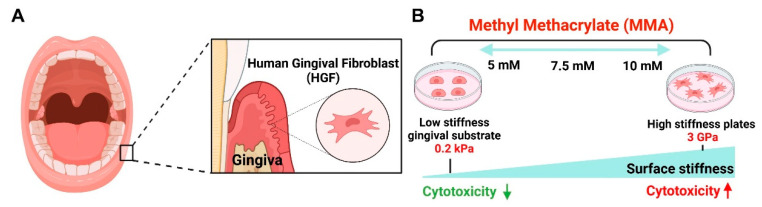
Schematic representation of substrate stiffness influencing MMA-induced cytotoxicity in HGFs. (**A**) This figure presents the effect of methyl methacrylate (MMA) on human gingival fibroblasts (HGFs) cultured on substrates of different stiffness (0.2 kPa and tissue culture plastic (TCP)). (**B**) MMA, a constituent of dental resins, generates toxic byproducts that impose cellular stress on gingival tissue. The schematic depicts a stiffness-dependent cytotoxic response, where increased substrate rigidity correlates with elevated cell death and DNA damage. Cells on the softer 0.2 kPa substrate exhibit reduced cytotoxicity compared to those on the stiffer TCP at MMA concentrations of 5 mM, 7.5 mM, and 10 mM.

In this study, we investigate the effect of substrate stiffness on the cytotoxicity of dental materials, focusing on methyl methacrylate (MMA). We compare the responses of human gingival fibroblasts (HGFs) cultured on soft, gel-based substrates that mimic oral tissue stiffness with those grown on conventional TCP. Notably, we selected a substrate stiffness of 0.2 kPa because, at this level, cells tend to adopt a rounder shape and are less spread out compared to cells on TCP (which has a stiffness of approximately 3 GPa). This morphological difference highlights the pronounced disparity between soft and stiff substrates, providing a better model for investigating the cytotoxic effects of MMA (Figure 1A,B). We hypothesize that cells on softer substrates will show greater resistance to cytotoxic stress, with higher viability, reduced apoptosis, and less DNA damage upon MMA, DMSO, and H_2_O_2_ exposure. By using a more physiologically relevant in vitro model, this study aims to bridge the gap between in vitro and in vivo conditions. Our findings could enhance the predictive accuracy of cytotoxicity assays and contribute to the development of more reliable biocompatibility testing protocols. This research may ultimately improve the evaluation of dental materials and guide the establishment of safer standards in clinical practice.

## 2. Materials and Methods

### 2.1. Cell Culture

Human gingival fibroblasts (HGFs) were isolated from gingival tissue collected during tooth extractions from patients in their twenties at the department of oral and maxillofacial surgery, Dankook University dental hospital (IRB No: DKUDH IRB 2023-06-001). Informed consent was obtained from all participants prior to sample collection. The fresh gingival tissue fragments were preserved in 7 mL of Hank’s balanced salt solution (HBSS, Welgene, Gyeongsan, Republic of Korea) containing 2% penicillin/streptomycin (PS; Thermo Fisher Scientific Inc., Waltham, MA, USA). After removing blood vessels using scissors and forceps, the fragments were digested in a solution containing 2 mg/mL collagenase type I and 4 mg/mL dispase II for 1 h at 37 °C in a water bath for dissociation. The gingival epithelial layer was separated, cut into approximately 1 × 1 mm^2^ pieces, and placed into 60 mm culture dishes (Falcon^®^, Corning, NY, USA). The tissues were cultured in minimum essential medium alpha modification (Alpha-MEM; LM008-01, Welgene) supplemented with 10% fetal bovine serum (FBS; Atlanta Biologicals, Flowery Branch, GA, USA), 2 mM L-glutamine (GlutaMAX™-1 100×; Gibco™, Thermo Fisher Scientific Inc.), 100 mM non-essential amino acid solution (100×; Gibco™), 55 μM 2-Mercaptoethanol (1000×; Gibco™), and 1% PS. The culture medium was refreshed after the first 24 h of incubation and subsequently every 2–3 days over the following 10–14 days. Primary cell colonies were collected and subcultured. The 6-well plates for TCP (#3006, SPL Life Science Co., Ltd., Pocheon-si, Republic of Korea) and 0.2 kPa substrates (#5165, Cytosoft^®^ 6-Well Plates, Advanced BioMatrix, Carlsbad, CA, USA) were coated with a solution of type I collagen (#5005, PureCol^®^, Advanced BioMatrix) and phosphate-buffered saline (PBS; Tech and Innovation, Chuncheon, Republic of Korea) at a 1:30 ratio for 2 h before seeding HGFs. Experiments were conducted 24 h post-seeding. HGFs at passages 8–10 were used for all experiments.

### 2.2. Substrate Stiffness Verification

The substrates used in this study, with stiffness values of 0.2, 2, 16, and 64 kPa, were obtained from Advanced BioMatrix. The stiffness values for each substrate were determined and certified by the manufacturer (Advanced BioMatrix). To ensure precise control over substrate stiffness, Advanced BioMatrix measured and validated the elastic modulus for each substrate during the manufacturing process using Atomic Force Microscopy (AFM) [32]. AFM-based measurements accurately determine the mechanical properties of substrates by recording responses to indentation depth and applied force, enabling precise elastic modulus calculations. These substrates, each featuring a 0.5 mm thick layer of biocompatible silicone gel, provide a range of physiologically relevant environments compared to stiff tissue culture plastic (TCP, 3 GPa), allowing us to investigate the effects of varying stiffnesses on cellular responses [33]. The layer of each substrate is functionalized to stably bind extracellular matrix (ECM) proteins, such as PureCol^®^ type I collagen (Advanced BioMatrix), to support stable cell adhesion. The thickness of the collagen coating layer is generally in the range of 30~50 nm and does not significantly affect the mechanical properties of the substrate [34,35,36]. All substrates were used as provided, without additional modifications.

### 2.3. Cell Viability

The cell viability of HGFs was assessed using the Cell Counting Kit-8 (CCK-8; CK04-20, Dojindo Laboratories, Kumamoto, Japan). After coating the wells of each plate for 2 h, HGFs were seeded at a density of 11 × 10^4^ cells/well for 0.2 kPa and 5 × 10^4^ cells/well for TCP. Following 24 h of incubation, cells were synchronized by replacing the medium with serum-free medium for 12 h. After this period, cells were treated with the respective solutions for 12 h, followed by the addition of 1 mL of 10% CCK-8 solution. Cells were then incubated at 37 °C for 2 h. After incubation, 100 µL of the supernatant from each well was transferred to a 96-well plate (#30096, SPL Life Science Co., Ltd.), and absorbance was measured at 450 nm using a microplate reader (Scientific Varioskan LUX Multimode Microplate Reader, Thermo Fisher).

### 2.4. Cell Morphology

To evaluate the effect of stiffness on the morphology of HGFs, cells were cultured on substrates with stiffness values of 0.2 kPa, 2 kPa, 16 kPa, 64 kPa (Advanced BioMatrix), and TCP (SPL Life Science). The 0.2 kPa substrate and TCP were both coated with type I collagen (Advanced BioMatrix) in PBS for 2 h, then incubated with cells at 37 °C for 24 h. After 24 h, cells were fixed with 4% paraformaldehyde (PFA; Tech & Innovation, Gangwon, Republic of Korea) at room temperature for 15 min and stained with DAPI (4′,6-diamidino-2-phenylindole, Thermo Fisher) and Phalloidin (Alexa FluorTM 488, Thermo Fisher). Stained images were captured using a fluorescence microscope (IX71, Olympus, Tokyo, Japan). Cell area, intensity, aspect ratio, and circularity were quantified using Image J software version 1.54d (National Institutes of Health, Bethesda, MD, USA) to compare differences in cell morphology.

### 2.5. Immunocytochemistry (ICC)

Immunocytochemistry (ICC) was performed to detect the expression of DNA damage markers. After culturing cells on 0.2 kPa and TCP, cells were fixed with 4% PFA (Tech & Innovation) at room temperature for 15 min. Following fixation, cells were washed three times with PBS for 5 min each, then permeabilized with PBS containing 3% bovine serum albumin (BSA; SolmateTM, Seoul, Republic of Korea) and 0.3% Triton^TM^ X-100 (Sigma-Aldrich, St. Louis, MO, USA) for 1 h. Cells were then incubated overnight at 4 °C with the primary antibody against phosphor-histone H2A.X (γH2AX; #9718, Rabbit mAb, Cell Signaling Technology, Danvers, MA, USA), a marker of DNA damage, diluted 1:400. After washing, samples were incubated at room temperature for 3 h with a fluorescein isocyanate (FITC)-conjugated anti-rabbit IgG secondary antibody (FITC; 711-025-152, Jackson ImmunoResearch, West Grove, PA, USA) at a 1:300 dilution. The cell nuclei were counterstained with DAPI (Thermo Fisher) for 10 min. Stained cells were imaged using a fluorescence microscope (IX71), and fluorescence intensity was quantified using ImageJ software (National Institutes of Health).

### 2.6. Effect of HGF Under Cytotoxicity Condition

Cell cytotoxicity assessments were conducted on both 0.2 kPa (Advanced BioMatrix) and TCP (SPL Life Science) substrates using dimethyl sulfoxide (DMSO; #D8418, Sigma-Aldrich)-induced toxicity; hydrogen peroxide (H_2_O_2_; H1009, Sigma-Aldrich)-induced oxidative stress; and methyl methacrylate (MMA; M55909, Sigma-Aldrich), a component of dental extracts. For all treatments, both the 0.2 kPa and TCP 6-well plates were coated with type I collagen (Advanced BioMatrix) in PBS for 2 h at room temperature. HGFs were seeded at a density of 11 × 10^4^ cells/well for the 0.2 kPa substrate and 5 × 10^4^ cells/well for TCP, followed by 24 h of incubation and a 12 h starvation period to synchronize the cell cycle. Cells were then treated with DMSO (1.25%, 2.5%, 5%, 10%, 20%), H_2_O_2_ (300 µM, 400 µM), or MMA (1 mM, 2.5 mM, 5 mM, 7.5 mM, 10 mM) for 12 h. Cell viability was assessed using the CCK-8 assay for each condition, and apoptosis was evaluated using Fluorescence-Activated Cell Sorting (FACS) analysis. For the MMA treatment, additional analyses included ICC to assess changes in cell morphology and DNA damage. Untreated cells served as the negative control for each treatment.

### 2.7. Flow Cytometry Analysis

Flow cytometry was performed to analyze apoptosis of HGFs in response to different substrate stiffnesses. Cells were seeded at densities of 11 × 10^4^ on 0.2 kPa and 5 × 10^4^ on TCP. After 24 h of incubation, cells were subjected to 12 h of starvation, followed by treatment with varying concentrations of DMSO (5% and 10%), H_2_O_2_ (300 μM and 400 μM), or MMA (5 mM, 7.5 mM and 10 mM) for 12 h. Cells were washed twice with cold PBS, incubated with trypsin-EDTA (Gibco™) for 3 min, and collected by centrifugation (3000 rpm, 3 min). The cell pellets were resuspended in 1× Annexin V Binding Buffer, which was prepared by diluting the 10× stock from the FITC Annexin V Apoptosis Detection Kit I (BD Biosciences, Franklin Lakes, NJ, USA) in distilled water at a 1:9 ratio. Cells were then incubated with 5 µL of FITC Annexin V and 5 µL of PI solution in the dark at room temperature for 15 min. After staining, 400 µL of 1× Binding Buffer was added to each sample, and apoptosis was analyzed using a BD Accury C6 plus flow cytometer (BD Biosciences). Quantification of FACS data was performed using FlowJo software v10.10. (BD Biosciences).

### 2.8. Statistical Analyses

Data from three independent experiments are shown, and statistical analyses were carried out using GraphPad Prism 8 software (San Diego, CA, USA). Statistical significance between groups was determined by two-way analysis of variance (ANOVA) with Tukey’s post hoc test for multiple comparisons and Student’s *t*-test. A *p*-value of <0.05 was considered statistically significant.

## 3. Results

### 3.1. Effect of Substrate Stiffness on HGF Morphology and Cell Spreading

**Figure 2:**
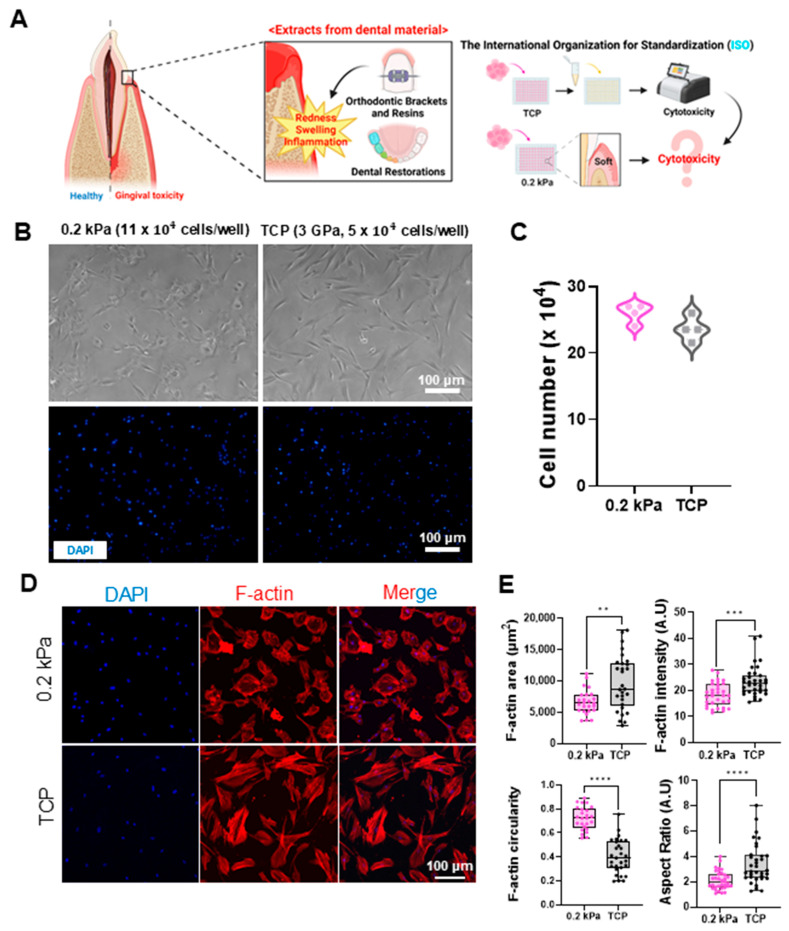
Impact of dental material extracts on gingival tissue and cellular responses to substrates with different stiffnesses. (**A**) Schematic representation of the potential cytotoxic effects of dental material extracts on gingival tissue. While current ISO standards utilize TCP (stiff) for testing, dental materials in actual gingival environments interact with soft tissues. This image illustrates the hypothesis that the cytotoxicity of these extracts may differ in soft gingival conditions compared to standard stiff substrates. (**B**) Representative bright-field (top) and DAPI-stained fluorescent images (bottom) of human gingival fibroblasts (HGFs) cultured on 0.2 kPa and TCP (3 GPa) substrates and seeded at 11 × 10^4^ cells/well and 5 × 10^4^ cells/well, respectively. Scale bar = 100 μm. (**C**) Quantitative comparison of HGF counts on 0.2 kPa and TCP after 24 h incubation, showing minimal differences (n = 4). (**D**) Immunofluorescent staining of DAPI (blue) and F-actin (red) in HGFs on each substrate. Scale bar = 100 μm. (**E**) Quantitative analysis of F-actin area, intensity, circularity, and aspect ratio. Cells on 0.2 kPa showed smaller areas, decreased intensity, and a more rounded morphology compared to TCP (n = 30). Data are mean ± SD from three independent experiments. Statistical significance was determined by Student’s *t-*test (** *p* < 0.01, *** *p* < 0.001, **** *p* < 0.0001).

Orthodontic brackets, dental resins, and restorations release substances such as methyl methacrylate (MMA) over time, which can induce inflammatory responses in gingival tissues, including redness and swelling [37,38,39,40]. Research indicates that MMA can leach from dental materials, reaching concentrations of 0.6 to 8.8 mg/L over 90 days and posing cytotoxic risks, especially when unpolymerized. Due to its cytotoxic, genotoxic, and inflammatory effects, as well as its gradual release under chewing forces, MMA raises concerns about potential long-term impacts on oral health [39,40]. Current ISO standards use stiff substrates like TCP (3 GPa) for cytotoxicity testing, while actual gingival tissue is much softer. This discrepancy raises concerns regarding the relevance of these standards for accurately assessing material toxicity in vivo. In this study, we examined the effects of MMA-containing dental material extracts on human gingival fibroblasts (HGFs) cultured on substrates with different stiffnesses (Figure 2A). Among the softer substrates tested, the 0.2 kPa condition exhibited the most distinct differences in cell shape, with cells displaying a more rounded morphology and reduced spreading compared to TCP. Therefore, this substrate stiffness was selected for subsequent detailed analysis (Figure S1A).

To maintain comparable growth conditions, we applied a higher initial seeding density on the 0.2 kPa matrix. Softer substrates, such as the 0.2 kPa gingiva-mimicking matrix, often provide reduced mechanical support, limiting initial cell adhesion and spreading, which can lead to slower proliferation compared to stiffer substrates like TCP [41,42,43]. To balance this difference and ensure similar cell confluence at the start of experiments, we increased the cell density on the softer substrate, enhancing cell–cell interactions and improving adhesion. This approach allowed us to directly compare cellular responses to cytotoxic treatments without the confounding variable of differing cell densities. As a result, we seeded 11 × 10^4^ cells on the 0.2 kPa substrate and 5 × 10^4^ cells on TCP, achieving similar cell counts after 24 h of incubation (Figure 2B,C). Cell morphology was then analyzed using immunocytochemistry (ICC). DAPI (blue) and F-actin (red) staining showed that cells on the 0.2 kPa were less spread out, with smaller cell areas and aspect ratios (AR), and had a more rounded morphology compared to cells on TCP (Figure 2D,E).

### 3.2. Substrate Stiffness Modulates DMSO-Induced Cytotoxicity in HGFs

**Figure 3:**
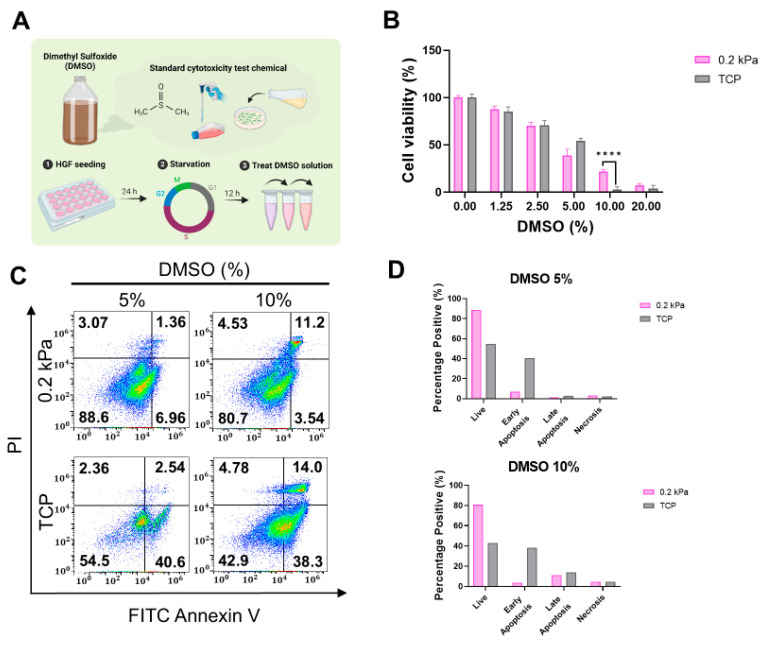
Effects of DMSO-induced cytotoxicity on HGFs cultured on 0.2 kPa and TCP. (**A**) Experimental workflow schematic for assessing DMSO cytotoxicity. DMSO, an organic solvent used to dissolve polar and non-polar compounds, serves as a standard cytotoxicity agent. HGFs were seeded for 24 h, starved for 12 h, then treated with varying DMSO concentrations. (**B**) Cell viability measured with Cell Counting Kit-8 (CCK-8) showed significantly higher viability at 10% DMSO on 0.2 kPa than on TCP. (**C**) Flow cytometry analysis displaying live, apoptotic, and necrotic cells after 5% and 10% DMSO treatment on both substrates. (**D**) Quantification of cell death distribution; live cell proportion was higher on 0.2 kPa, while apoptotic cells were more prevalent on TCP. Representative data sets after three independent experiments are shown. Statistical significance was determined by two-way analysis of variance (ANOVA) with Tukey’s post hoc test for multiple comparison (**** *p* < 0.0001).

We first assessed the cytotoxicity of dimethyl sulfoxide (DMSO), a chemical widely used in standard cytotoxicity tests, on substrates with stiffnesses of 0.2 kPa and TCP [44]. Various concentrations of DMSO were applied to assess its cytotoxic effects (Figure 3A). As shown in Figure 3B, cell viability was comparable between the 0.2 kPa and TCP conditions after DMSO treatment, although the viability was higher on the 0.2 kPa substrate at 10% DMSO. Flow cytometry analysis supported this result, revealing a greater proportion of live cells on the 0.2 kPa substrate, while early and late apoptosis rates were elevated on TCP (Figure 3C,D). These findings suggest that the softer substrate may provide a protective effect against DMSO-induced apoptosis compared to TCP.

### 3.3. Substrate Stiffness Modulates Cellular Resistance to H_2_O_2_-Induced Oxidative Stress

**Figure 4:**
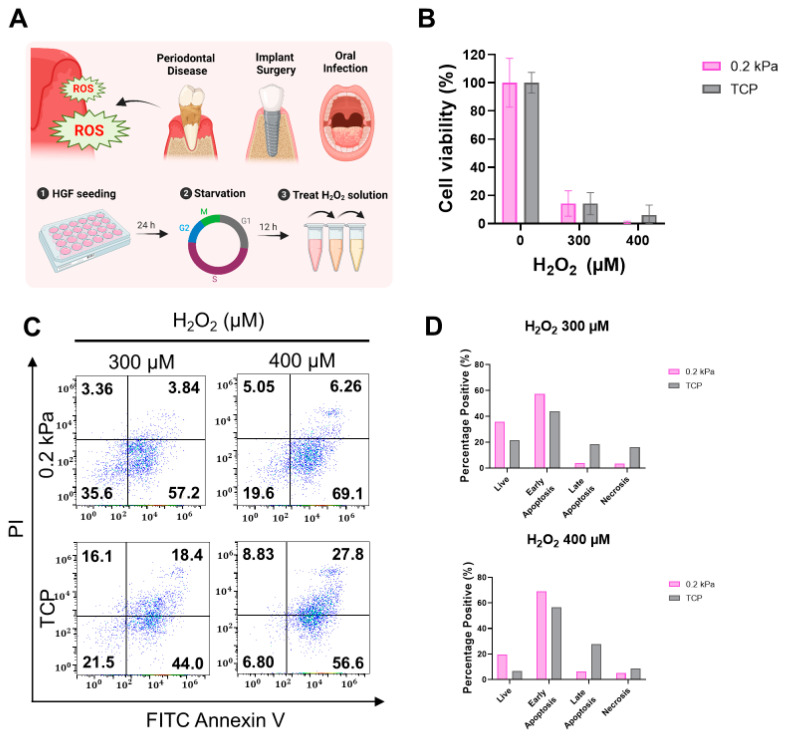
Effects of H_2_O_2_-induced oxidative stress on HGFs cultured on 0.2 kPa and TCP. (**A**) Schematic of the experimental design highlighting the role of reactive oxygen species (ROS) in periodontal disease, implants, and infections. Human gingival fibroblasts (HGFs) were seeded for 24 h on 0.2 kPa and TCP, starved for 12 h, and treated with hydrogen peroxide (H_2_O_2_). (**B**) Cell viability after treatment with 300 µM and 400 µM H_2_O_2_ on both substrates, measured with Cell Counting Kit-8 (CCK-8), showing minimal differences. (**C**) Flow cytometry displaying the distribution of live, apoptotic, and necrotic cells after H_2_O_2_ treatment. (**D**) Quantification of cell death distribution; live cells and early apoptosis were higher on 0.2 kPa, while late apoptosis and necrosis were more pronounced on TCP. Representative data sets from three independent experiments are shown in the manuscript.

Next, we evaluated the effects of hydrogen peroxide (H_2_O_2_)-induced oxidative stress on the 0.2 kPa and TCP. After seeding, the cells were subjected to a 12 h starvation period to synchronize the cell cycle. HGFs were then treated with H_2_O_2_ at concentrations of 300 µM and 400 µM to assess cytotoxicity (Figure 4A). As shown in Figure 4B, cell viability showed no significant difference between the 0.2 kPa and TCP at both concentrations. To further investigate cell viability, flow cytometry analysis was performed. Flow cytometry analysis revealed higher levels of late apoptosis and necrosis in cells cultured on TCP, particularly at 400 µM H_2_O_2_. In contrast, a higher proportion of live cells was observed on 0.2 kPa. Quantification of apoptotic and necrotic populations (Figure 4C,D) confirmed that 0.2 kPa provided greater protection against H_2_O_2_-induced apoptosis and necrosis. These results suggest that substrate stiffness influences cellular responses to oxidative stress, with softer substrates offering enhanced resistance to H_2_O_2_-induced cytotoxicity.

### 3.4. Matrix Stiffness Modulates HGF Responses to MMA-Induced Cytotoxicity and DNA Damage

**Figure 5:**
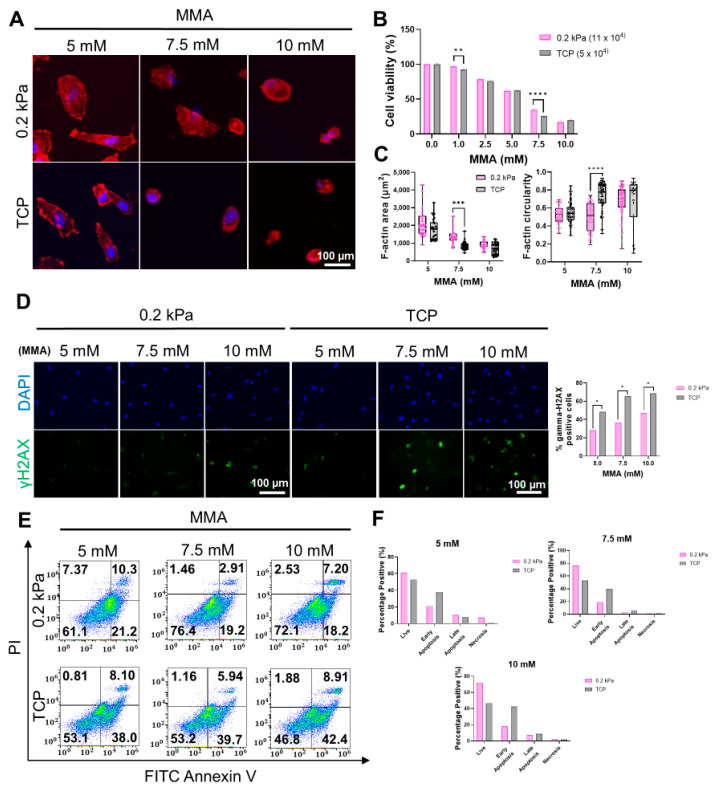
Effects of MMA on HGFs cultured on 0.2 kPa and TCP. (**A**) Immunofluorescence images of DAPI (blue) and F-actin (red)-stained HGFs treated with 5 mM, 7.5 mM, and 10 mM MMA for 12 h, showing cell morphology differences between 0.2 kPa and TCP substrates. Scale bar = 100 µm. (**B**) Cell viability after MMA treatment, indicating a significant reduction in TCP compared to 0.2 kPa at higher MMA concentrations (n = 5). (**C**) Quantification of cell area and circularity, showing a smaller cell area and more spreading on 0.2 kPa (n = 90). (**D**) Immunofluorescence staining for γH2AX (green) and DAPI (blue) showing DNA damage; TCP cells exhibited higher γH2AX levels. Scale bar = 100 µm. (**E**) Flow cytometry analysis of apoptosis and necrosis following MMA treatment, with higher apoptotic/necrotic populations on TCP. (**F**) Quantification of apoptotic and necrotic cells, with apoptosis and necrosis increasing at higher MMA concentrations, particularly on TCP. Representative data sets after three independent experiments are shown. Statistical significance was determined by two-way ANOVA followed by Tukey’s multiple comparison test (ns = non-significant, * *p* < 0.05, ** *p* < 0.01, *** *p* < 0.001, **** *p* < 0.0001).

To validate our hypothesis, we exposed HGFs to MMA at concentrations of 5, 7.5, and 10 mM and evaluated the effects on cell viability, morphology, DNA damage, and apoptosis/necrosis. ICC was employed to assess cellular morphology, revealing that cells cultured on 0.2 kPa exhibited significantly higher viability at all MMA concentrations compared to TCP, with a particularly marked difference at 7.5 mM (Figure 5A,B). Cells on 0.2 kPa also showed enhanced spreading and elongation, in contrast to the more rounded morphology observed on TCP (Figure 5C). Next, we investigated the impact of substrate stiffness on DNA damage induced by MMA. As shown in Figure 5D, γH2AX foci were significantly more frequent in cells on TCP compared to those on 0.2 kPa, with this effect becoming more pronounced at higher MMA concentrations. To further clarify these observations, flow cytometry analysis was conducted, which confirmed a higher proportion of live cells on 0.2 kPa across all MMA concentrations. In contrast, cells cultured on TCP exhibited significantly elevated levels of apoptosis (Figure 5E,F). These results suggest that matrix stiffness critically modulates cellular responses to MMA-induced stress, with softer substrates conferring enhanced protection against cytotoxicity, DNA damage, and apoptosis.

## 4. Discussion

In the context of current ISO standards for cytotoxicity testing, stiff tissue culture plastic (TCP) is predominantly used as a substrate for in vitro experiments [9]. However, TCP, with its stiffness of 3 GPa, fails to replicate the mechanical properties of soft tissues such as the gingiva, which exhibit much lower stiffness. This discrepancy can lead to incomplete or inaccurate assessments of cellular responses to cytotoxic agents, particularly when evaluating materials that interact with soft tissues, such as dental resins containing methyl methacrylate (MMA). By employing a 0.2 kPa substrate that closely approximates the mechanical stiffness of human gingival tissue, we sought to better mimic the native in vivo environment and explore how substrate stiffness influences the cytotoxic effects of MMA on human gingival fibroblasts (HGFs).

**Figure 6:**
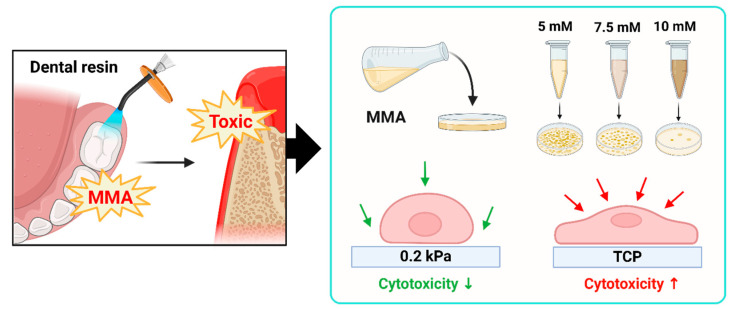
Schematic overview of surface stiffness and cytotoxicity effects of MMA on gingival tissue. Dental resin materials containing methyl methacrylate (MMA) can release toxic substances, leading to gingival irritation and inflammation. This figure illustrates the relationship between surface stiffness and cytotoxicity in human gingival fibroblasts (HGF) exposed to increasing concentrations of MMA (5 mM, 7.5 mM, 10 mM) on 0.2 kPa and TCP substrates. As surface stiffness increases, cytotoxicity is elevated, suggesting a stiffness-dependent cytotoxic response to MMA exposure.

Our findings highlight the significant role that substrate stiffness plays in modulating cellular responses to cytotoxic stress. HGFs cultured on the softer 0.2 kPa substrate exhibited enhanced resistance to MMA-induced cytotoxicity, as evidenced by higher cell viability, reduced apoptosis, and less DNA damage, compared to cells cultured on the stiffer TCP. These results suggest that softer substrates, which better replicate the biomechanical properties of gingival tissue, may confer a protective effect against MMA toxicity. This observation is supported by the fact that cells on softer substrates displayed a more rounded morphology, decreased cell spreading, and smaller F-actin areas, indicative of reduced mechanical stress. In contrast, cells cultured on TCP showed more pronounced stress responses, including larger cell areas, more spread morphology, and higher levels of F-actin intensity (Figure 6).

One potential explanation for the enhanced resistance to cytotoxicity observed on the softer substrate is the difference in the surface area-to-volume ratio between cells grown on 0.2 kPa and those grown on TCP. Cells on softer substrates tend to adopt a smaller, more rounded morphology, which may reduce the surface area that is exposed to toxic agents like MMA [45,46]. This, in turn, could limit the amount of MMA that can penetrate or interact with the cells, thus reducing the cytotoxic impact [47]. Additionally, the softer matrix may better support the cells’ intrinsic repair mechanisms, helping to mitigate DNA damage [48,49,50], as evidenced by the lower frequency of γH2AX-positive cells on the 0.2 kPa substrate compared to TCP. These findings align with previous studies suggesting that smaller cells with less spread may be less susceptible to cytotoxic damage due to lower toxin exposure and penetration.

Flow cytometry analysis further supported these observations, showing that cells cultured on the 0.2 kPa substrate had higher viability and lower apoptosis and necrosis rates, particularly at higher MMA concentrations. In contrast, cells on TCP exhibited significantly higher levels of apoptosis and necrosis, particularly in the late stages of apoptosis. These data suggest that the softer substrate not only reduces the initial cytotoxic impact, but also enhances cellular resilience to prolonged exposure to MMA.

Moreover, a differential response of HGFs to MMA-induced oxidative stress was also observed. Cells cultured on the softer substrate showed less oxidative stress-induced apoptosis and necrosis in response to hydrogen peroxide (H_2_O_2_), further demonstrating the protective effect of a physiologically relevant substrate. This finding is particularly relevant given that oxidative stress is a common mechanism of cytotoxicity in gingival tissue exposed to dental materials over time [51,52,53].

These findings challenge the adequacy of current ISO standards, which rely on stiff substrates like TCP for assessing the cytotoxicity of materials intended for soft tissue applications. Our results suggest that incorporating more physiologically relevant substrates, such as the 0.2 kPa matrix used in this study, would provide a more accurate reflection of in vivo behavior, particularly for materials like MMA that interact with soft tissues. Further research is needed to understand why cells on softer substrates, which tend to be less spread, may exhibit increased resistance to cytotoxic damage. One hypothesis is that less cell spreading results in lower toxin exposure and penetration due to the reduced surface area for interaction. Investigating the underlying mechanisms behind this difference could shed light on how substrate stiffness influences cellular sensitivity to toxins, leading to more accurate cytotoxicity assessments and improved material safety for clinical use.

## 5. Conclusions

Our findings suggest that substrate stiffness may influence the cytotoxic response of HGFs to MMA, although broader studies are needed to confirm this effect across various conditions. Using a 0.2 kPa substrate provided a more physiologically relevant model compared to conventional stiff TCP, supporting the need to revise current cytotoxicity testing standards to include softer substrates that better reflect the in vivo environment, thereby enhancing the safety assessment of dental materials. However, testing only two stiffness levels limits our comprehensive understanding of how substrate mechanics affect cellular responses. Future studies should, therefore, investigate the molecular mechanisms underlying the potential protective effects of soft matrices and evaluate their relevance across various cytotoxic agents and soft tissues. Additionally, validating these findings in 3D or in vivo models could further improve the accuracy of cytotoxicity assessments for soft tissue applications.

## Data Availability

The original contributions presented in this study are included in the article/Supplementary Material. Further inquiries can be directed to the corresponding author(s).

## Supplementary Materials

The following supporting information can be downloaded at: https://www.mdpi.com/article/10.3390/cells13231932/s1, Figure S1: HGF Morphological Changes on Substrates with different Stiffness.
