# Supplementary material for: Influence of Soft and Stiff Matrices on Cytotoxicity in Gingival Fibroblasts: Implications for Soft Tissue Biocompatibility
Source: Cells. 2024 Nov 21;13(23):1932. doi: 10.3390/cells13231932 (PMC11639834; doi:10.3390/cells13231932)
Supplement: Supplementary file 1 [file cells-13-01932-s001.zip › cells-3290842-supplementary.pdf]

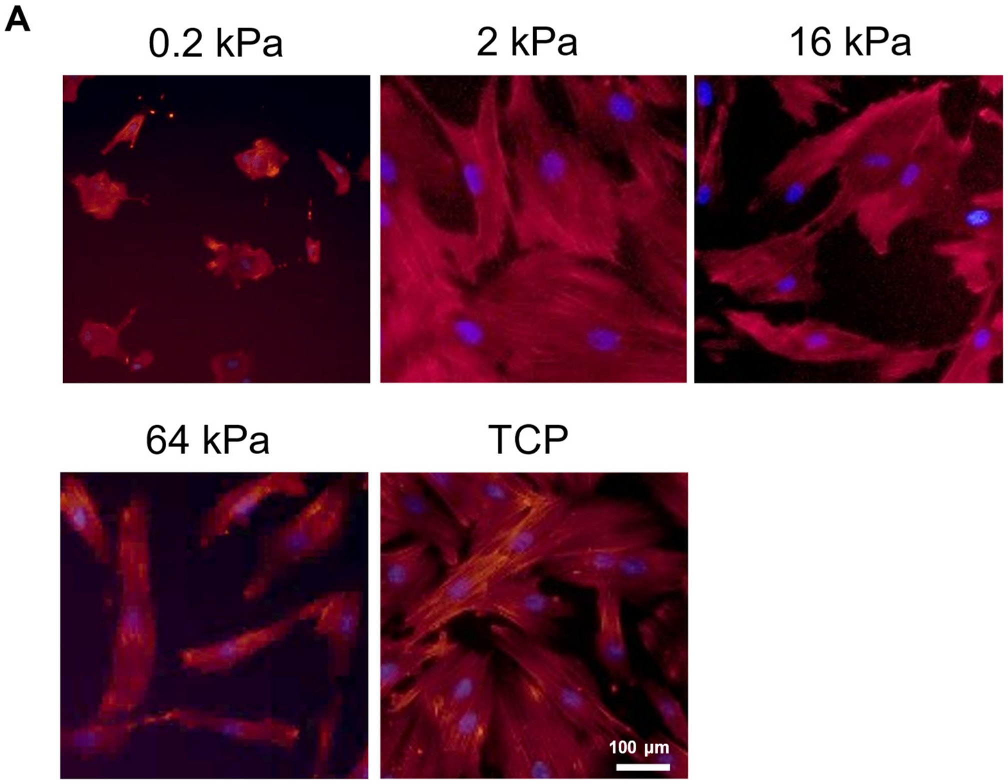

Figure S1. HGF Morphological Changes on Substrates with different Stiffness. (A) Immunofluorescent staining of DAPI (blue) and F-actin (red) in HGFs cultured on 0.2 kPa, 2 kPa, 16 kPa, 64 kPa and TCP. Distinct morphological differences were observed, with cells on the 0.2 kPa exhibiting a more rounded and less spread shape, while cells on the 2 kPa, 16 kPa, 64 kPa and TCP appeared more elongated and spread out. Based on these observations, 0.2 kPa and TCP were selected for further analysis to investigate the effects of substrate stiffness on cell morphology and behavior. Scale bar = 100 μm.
